# Long-Duration Carbon Dioxide Anesthesia of Fish Using Ultra Fine (Nano-Scale) Bubbles

**DOI:** 10.1371/journal.pone.0153542

**Published:** 2016-04-21

**Authors:** Kenji Kugino, Shizuka Tamaru, Yuko Hisatomi, Tadashi Sakaguchi

**Affiliations:** 1 Division of Nutritional Science, Graduate School of Human Health Science, University of Nagasaki, Nagasaki, 851–2195, Japan; 2 Department of Health and Nutrition Science, Faculty of Health and Social Welfare Science, Nishikyushu University, Saga, 842–0015, Japan; 3 Institute of Marine Sciences and Technologies, Marine Biotechnology Inc, Fukuoka, 811–3501, Japan; University of Bari, ITALY

## Abstract

Introduction: We investigated whether adding ultrafine (nano-scale) oxygen-carrying bubbles to water concurrently with dissolved carbon-dioxide (CO_2_) could result in safe, long-duration anesthesia for fish. Results: To confirm the lethal effects of CO_2_ alone, fishes were anesthetized with dissolved CO_2_ in 20°C seawater. Within 30 minutes, all fishes, regardless of species, died suddenly due to CO_2_-induced narcosis, even when the water was saturated with oxygen. Death was attributed to respiration failure caused by hypoxemia. When ultrafine oxygen-carrying bubbles were supplied along with dissolved CO_2_, five chicken grunts were able to remain anesthetized for 22 hours and awoke normally within 2–3 hours after cessation of anesthesia. Conclusions: The high internal pressures and oxygen levels of the ultrafine bubbles enabled efficient oxygen diffusion across the branchia and permitted the organismal oxygen demands of individual anesthetized fish to be met. Thus, we demonstrated a method for safe, long-duration carbon dioxide anesthesia in living fish under normal water temperatures.

## Introduction

In the field of fish culturing, anesthetic agents for animals are commonly used in vaccination, labeling, and various measurements [1–2]. But no agents have been able to achieve long-duration anesthesia in fishes. The use of traditional anesthetic agents for animals in the fishery is not desirable in terms of environmental protection because the discarded solutions directly enter oceans and rivers. The use of anesthetic agents that pose a risk of remaining in the tissues of cultured fish has been cautioned against. For example, the Japanese Ministry of Agriculture, Forestry, and Fisheries cautions against the use of anesthetic agents within 7 days before fishes and shellfishes are catched.

The method of quiescent fish by immersion in cold water with no anesthetic agent has been approved [2–4], but it is not widely used due to the need to install large refrigeration systems, which consume large quantities of electrical power. Another widely known method for short-duration anesthesia uses carbon dioxide dissolved in water [5–6]. Carbon dioxide has anesthetic effects in various terrestrial and aquatic animals. Because toxic substrates do not remain in these creatures, carbon dioxide is an ideal anesthetic for organisms intended as human food sources. However, carbon dioxide anesthesia has limited application in aquatic creatures because it quickly induces sudden death due to respiratory failure, even if given for short periods [7]. Development of long-duration quiescent technology utilizing carbon dioxide would be of wide application and high utility in fields dealing with aquatic organisms.

Rate of oxygen diffusion into the branchial capillaries of fish depends on the difference in gas partial pressures ([partial pressure of oxygen dissolved in water]—[partial pressure of oxygen dissolved in branchial capillary]). When fish are anesthetized, muscle movements in the respiratory branchia are suppressed and oxygen uptake by diffusive transfer decreases. This results in oxygen deficiency relative to organismal oxygen demand, which leads to a sudden death, even if the creature is placed in water saturated with dissolved oxygen. Therefore, in order to achieve long-duration anesthesia for fish and shellfish, either organismal oxygen demand must be lowered or increased dissolved oxygen must be supplied via oversaturation of the aquatic environment.

Methods to decrease oxygen demand include inducing artificial hibernation [8], using cold carbon dioxide under low temperatures [9], and using an anesthetic apparatus under low temperatures (a more accurate method) [10]. However, these methods are problematic. Habituating fish and shellfish to low temperature (5°C) requires several days and, therefore, the installation of a large refrigeration system. Once cold-water methods are excluded, the alternative of supplying an oversaturated dissolved oxygen environment remains.

In this study, we investigated the use of carbon dioxide under oxygenation with ultrafine (nano-size) bubbles as a long-duration anesthetic.

## Materials and Methods

First, we confirmed the time limit for conventional carbon dioxide anesthesia for fishes. Fish species and number of samples used for the experiment are listed in Table 1. A circular tank of 400 L capacity held seawater maintained at 20°C. Oxygen and carbon dioxide were aerated into the seawater using a generic air pump and air stone. The concentration of dissolved carbon dioxide was raised incrementally at a rate of 0.5% per minute, until fishes were anesthetized under a saturated dissolved oxygen environment. Anesthesia initiation was estimated to have occurred when body movement (swimming behavior), excluding respiratory action of the branchia, ceased, as seen in a monitoring camera. At that point, anesthesia was continued at a slightly higher level of carbon dioxide. Vitality was determined every 5 minutes and mortality was determined to have occurred when branchial movement was no longer detected. The carbon dioxide level of seawater was measured with a meter (type CGP-31, TOADKK Co. Ltd, Japan) and described in terms of v/v%.

**Table 1:** Various aquatic species and number of samples used for the experiment of conventional carbon dioxide-anesthesia,

Fish species	Number of samples
Bigfin reef squid (*Sepioteuthis lessoniana*)	2
White-lined rockcod (*Anyperodon leucogrammicus*)	2
Chicken grunt (*Parapristipoma trilineatum*)	2
Japanese scad (*Trachurus japonicas*)	2
Red seabream (*Pagrus major*)	2

Secondly, we investigated long-duration anesthesia using carbon dioxide with an oversupply of oxygen using five chicken grunts, weighing about 450 g each, and the same apparatus as in the earlier experiment, under otherwise similar conditions. In this case, ultrafine oxygen-carrying bubbles were supplied continuously throughout the process via a nano bubble generator (type IOHOK2014, Hanagiken Co. Ltd, Japan) that could stably generate ultrafine bubbles; the diameters are less than 1 μm (around 200–300 nm). The final concentration of the ultrafine bubbles was 30 million per mL of seawater. Carbon dioxide was aerated into the seawater and increased incrementally to 6.0%. Visualization via monitoring camera confirmed that all chicken grunts stopped body movement (swimming behavior), except for respiratory action of branchia, when the carbon dioxide level reached 6.0%. At that point, carbon dioxide was maintained at a level of 5.0% for a period lasting 22 hours. After anesthesia, oxygen was aerated into seawater using a generic air pump and air stone, thereby lowering the carbon dioxide level gradually at a rate of 1% per 30 minutes to awaken the chicken grunts.

This study was approved by the Animal Use Committee of Marine Biotechnology Inc. (approval number, 14–01) and was carried out as per the guidelines for animal experiments of Marine Biotechnology Inc. and Law No. 105 and Notification No. 6 of the Government of Japan.

## Results

The time limits for conventional carbon dioxide anesthesia for various aquatic species are shown in Table 2. Carbon dioxide levels for anesthesia initiation and for maintenance of anesthesia were 2.9–8.9% and 5–10%, respectively, in all species. All species anesthetized with a high level of carbon dioxide died suddenly within 25 minutes of anesthesia initiation. We were able to observe behaviors indicating struggle (i.e., fish opened mouths and opercula wide, several times) just before death was confirmed by visualization, due to lowered respiratory exercise, and the color of capillary in branchia was dark red, not bright red when observed in living fishes. Thus, we confirmed that fishes and invertebrates, regardless of species, died suddenly with carbon dioxide anesthesia, even if placed in oxygen-saturated water.

**Table 2:** The time limits for conventional carbon dioxide-anesthesia for various aquatic species.

Fish species	Individual number	CO_2_ level for anesthesia initiation* (%)	CO_2_ level for maintenance of anesthesia (%)	Lethal time(min.)
Bigfin reef squid	No.1	5.3	7.0	10
	No.2	4.2	7.0	15
White-lined rockcod	No.3	6.8	8.0	10
	No.4	5.8	8.0	20
Chicken grunt	No.5	4.0	5.0	10
	No.6	2.9	5.0	10
Japanese scad	No.7	4.3	7.0	10
	No.8	6.0	7.0	15
Red seabream	No.9	8.9	10.0	25
	No.10	7.3	10.0	15

* Carbon dioxide concentration judged by macroscopic observation when it was in the anesthetic condition that the condition of the fish was equivalent to for the second phase from the first phase of the depth of anesthesia in human general anesthesia.

**Fig 1:**
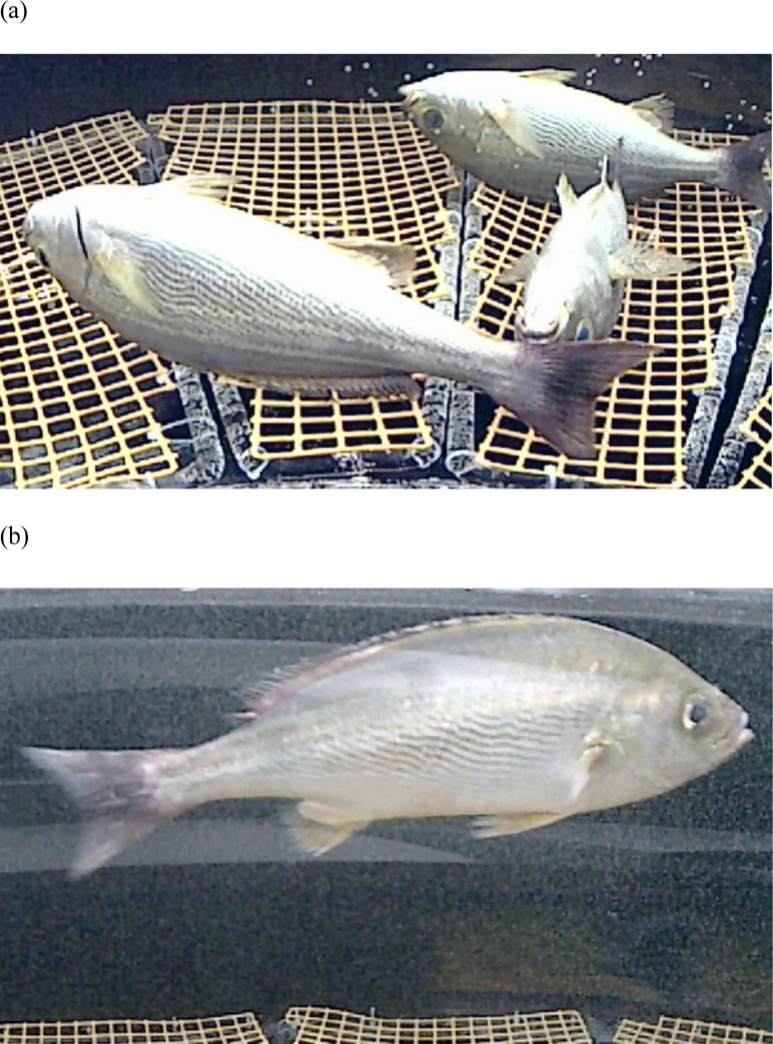
Chicken grunts anesthetized with carbon dioxide under water oxygenated with fine bubbles. (a) All chicken grunts were anesthetized. (b) After 22 hours under anesthesia, carbon dioxide level was lowered and chicken grunts started to awaken and swim normally within 2–3 hours.

Next, chicken grunts were anesthetized with carbon dioxide under water oxygenated with fine bubbles. As shown in Table 3, carbon dioxide levels at anesthesia initiation were 2.8–4.2%. After anesthesia was maintained by a carbon dioxide level of 5.0% for 22 hours, carbon dioxide was lowered to 1.2–2.0% and the chicken grunts awoke. The process is shown in Fig 1. All chicken grunts were anesthetized, and the branchial and respiratory exercise were normal (a). After 22 hours under anesthesia, carbon dioxide level was lowered and chicken grunts started to awaken and swim normally within 2–3 hours (b). No abnormal fishes were observed after 24 hours of awaking. Thus, we were able to demonstrate that when dissolved carbon dioxide is supplied concurrently with ultrafine oxygen-carrying bubbles, fishes can be anesthetized safely for long periods.

**Table 3:** Carbon dioxide-anesthesia under water oxygenated with ultrafine bubbles.

Fish species	Individual number	CO_2_ level at anesthesia initiation* (%)	CO_2_ level at the maintenance of anesthesia (%)	CO_2_ level at anesthesia awakening (%)
Chicken grunts	No.1	2.8	5.0	1.2
	No.2	3.8	5.0	1.8
	No.3	3.0	5.0	1.2
	No.4	3.4	5.0	1.2
	No.5	4.2	5.0	2.0

* Carbon dioxide concentration judged by macroscopic observation when it was in the anesthetic condition that the condition of the fish was equivalent to for the second phase from the first phase of the depth of anesthesia in human general anesthesia.

## Discussion

Carbon dioxide has been known to produce an anesthetic effect in a wide variety of terrestrial and aquatic creatures. In contrast to general anesthetic agents, the use of carbon dioxide ensures that no toxic substrates remain in anesthetized creatures, an important consideration when the animals also serve as sources of food. Therefore, carbon dioxide is recommended for edible creatures and has been used widely in the preparation of killing of farm animals. However, many reports suggest that, without exception, aquatic creatures anesthetized with carbon dioxide die suddenly within a short period (within twenty minutes); therefore, the application of carbon dioxide as an anesthetic has been limited [5–7]. We were unable to find reports detailing the mechanism of sudden death from carbon dioxide. The sudden death was thought to be not due to anoxia, because increased dissolved oxygen (100% of dissolved oxygen) was maintained via aeration when fish and shellfish was anesthetized.

In contrast, FA100, containing mainly eugenol, is approved for medicinal use in animals and has been used as an anesthetic agent for fish and shellfish in Japan and other countries [11]. FA100 is used widely for injection of vaccine and sex discrimination. However, FA100 is used for short-duration anesthesia lasting no more than several minutes, and in the case of use over 20 minutes, FA100 causes death [11]. Non-approved anesthetic agent, 2-phenoxyethanol, is reported to be more likely to recover from anesthesia than FA100, but the anesthetic effect is equivalent to FA100. Furthermore, the application possibility of carbon dioxide as anesthetic agent has been investigated, which has been concluded that carbon dioxide is a great method in short time-anesthesia but cannot be used for long time-anesthesia [12–13]. So until now, no safe, convenient, and fully effective method for long-term anesthesia of fish and shellfish has existed.

In animals, including humans, the most frequent complication associated with anesthesia is respiratory failure due to anoxia. When the animals are anesthetized, high levels of oxygen must be inhaled to prevent respiratory failure caused by reduced ventilation volume in lung. In clinical settings, the concentration of supplied oxygen is within the range of 40–80% (2–4 fold that of normal air). Oxygen uptake at the terminal (alveolus) of the respiratory organ (lung) is determined by the differences in oxygen partial pressures between the alveolus and the surrounding capillary. The oxygen uptake required for a living organism can be achieved by enhancing the difference between internal and extracorporeal gas partial pressures, which leads to an increase in oxygen uptake into the capillary and compensates for a lowered rate of respiratory exercise due to anesthesia.

The lung respiratory exercise of terrestrial creatures at rest is able to meet oxygen demand under atmospheric oxygen concentrations (approximately 21%). When exertive physical activity must be performed for predation or escape, the increased oxygen demand rapidly results in an oxygen debt, which can be resolved by increasing respiratory exercise to supplement the depleted oxygen. Animals normally maintain sufficient respiratory function to meet oxygen demands at rest. However, the fact that an oxygen debt is quickly incurred when performing physical activity indicates that the spare capacity of the respiratory apparatus, relative to the resting state, is not generally high. Therefore, to avoid respiration failure during anesthesia, lowered gas exchange-efficiency is should be compensated by providing higher levels of oxygen.

In contrast to terrestrial vertebrates, fish and shellfish take up dissolved oxygen by branchial respiration. The capillary in the branchial terminal (secondly branchial filament) makes contact with water, but has the same basic structure as the human alveolus (i.e., oxygen diffuses due to the concentration gradient between the water and the capillary blood). The difference between lung and branchial respiration is only in whether alveolar surface or water is in contact with the capillary. Marine surveys reveal that in the waters in which most fish and shellfish are found (at depths less than 200 meters), oxygen levels are at 70–100% saturation (8.84 mg/L at 20°C) [14]. In other words, fish and shellfish are adapted to aquatic environments at or very close to the maximum possible concentration of oxygen. When anesthetized, dissolved oxygen is usually maintained at 100% saturation and a higher oxygen level cannot be provided if oxygen is supplied merely by aeration. Conventional knowledge suggests that oxygenation under anesthesia is enough because dissolved oxygen is saturated, and the lack of oxygen supplied to the organism cannot occur under sufficient aeration. However, most fish and shellfish necessarily fall into hypoxemia under anesthesia even in conditions of 100% dissolved oxygen, because these creatures are living and adapting to oxygen-saturated waters. In this study, all subjects died suddenly within 30 minutes of after carbon dioxide anesthesia in water with 100% dissolved oxygen as shown in Table 2. And we could observe the signs of struggle (opening the mouth and opercula widely several times) just before death, due to lowered respiratory exercise. That is, when respiratory exercise is suppressed, the oxygen diffusion resulting from differences in partial pressures between the water and branchial capillaries decreases, and oxygen uptake is reduced, resulting in death due to hypoxemia [5–7]. A long-duration anesthesia for fish and shellfish requires the provision of an aquatic environment in which the level of dissolved oxygen is higher than that of the usual aquatic environment (100% dissolved oxygen), but in practice it is difficult to achieve oxygen supersaturation.

Thus, we utilized ultrafine bubbles that are able to remain suspended in water, rather than dissolved oxygen, as the method of oxygenation. The diameter of the bubbles in water is reflected in buoyancy and rising rate. The rising rate depends on solution properties, and Reynolds number (Re) corresponds to approximately 1 at about 100 μm of diameter. In addition, in the case of Re < 1, Stoke’s law adapts well because bubbles behave as balls due to flux conditions on interface of globular bubbles [15]. Results of measurements using distillated or tap water are consistent with the value calculated by Stoke’s law [15]. Table 4 indicates the rising rate calculated using Stoke’s law. In this result, bubbles with diameters under 1 μm (ultrafine bubbles) remain in water without surfacing for an hour. The relationship between bubble diameter and pressure is given by the Young-Laplace equation as ΔP = 4σ/d [16–17]. Table 5 shows the calculations derived using the Young-Laplace equation with values for surface tension of σ = 72.8 mN/m (20°C) and pressure surrounding the bubble of 1 atm. Internal pressures of bubbles smaller than 1 μm diameter range from several to dozens of times larger than atmospheric pressure. These physical properties suggest the possibility of using ultrafine bubbles as oxygen-carrying bodies.

**Table 4:** Relationship with the bubble diameter and the rising rate of a bubble in water.

Diameter of bubble	Rising rate of bubble in water (v_s_)
100 μm	5440 μm/s
10 μm	54.4 μm/s ≒ 19.6 cm/h
1 μm	0.544 μm/s ≒ 2.0 mm/h

Stokes' law is as follows: [v_s_ = D_p_^2^ (σ_p_−σ_f_) g] / 18η

v_s_: Terminal velocity [cm/s], D_p_: Particle diameter [cm], σ_p_: Particulate density [g/cm^3^], σ_f_: Density of the fluid [g/cm^3^], g: Acceleration of gravity [cm/s^2^], η: Coefficient of viscosity of the fluid [g/(cm·s)].

**Table 5:** Relationship with the bubble diameter and the pressure in the bubble.

Diameter of bubble	Pressure in the bubble in water
1 mm	1.003 atm
100 μm	1.03 atm
10 μm	1.29 atm
1 μm	3.9 atm
500 nm	5.8 atm
300 nm	9.7 atm
200 nm	14.6 atm
100 nm	29.7 atm

Young-Laplace equation is as follows: ΔP = 4σ/d

ΔP: Degree of the upward pressure [atm], σ: Surface tension [mN/m], d: Diameter of bubble [mm].

By supplying ultrafine oxygen-containing bubbles to the aquatic environment, we achieved a higher level of oxygenation, allowing us to develop a method for avoiding hypoxemia during anesthesia of fish and shellfish. As a result, we discovered that it is possible to achieve long-duration anesthesia (22 hours) with carbon dioxide in chicken grunt (Table 1, Fig 1). In this experiment, carbon dioxide was dissolved into water by aeration. Sufficient anesthetic depth (equivalent to the first to second phase of human general anesthesia, or anesthesia of the thalamus, cortex nucleus, and spinal cord) was achieved to still the chicken grunt.

Meanwhile, oxygen-carrying ultrafine bubbles were continuously and stably transported to the branchia of fish by water flow. The ultrafine bubbles had sufficient pressure and oxygen for efficient diffusion of oxygen across branchia, successfully meeting organismal oxygen demand during anesthesia despite the limited volume occupied by the bubbles. Oxygen uptake via branchia lamella capillary changes according to the diffusion coefficient, and depends on the diameter (internal pressure) and number of ultrafine bubbles contacting the membrane surface of branchial epithelial cells. That is, we presume that alteration of the ultrafine bubbles supplied to the water can be adjusted to meet the oxygen demands of particular fish species.

The applications of this method in diverse areas will be an agenda for future research. For example, the transport of living fish over long periods and distances, removal of blood or other medical interventions in large fish, and uptake of medication by cultured fish may all be enhanced by the application of ultrafine bubble technology.

## Conclusions

We demonstrated a method for safe and long-duration carbon dioxide anesthesia in living fish under normal water temperatures by using ultrafine (nano-scale) bubbles as the oxygenation bodies.

## Supporting Information

S1 Table The time limits for conventional carbon dioxide-anesthesia for various aquatic species. * Carbon dioxide concentration judged by macroscopic observation when it was in the anesthetic condition that the condition of the fish was equivalent to for the second phase from the first phase of the depth of anesthesia in human general anesthesia.
(DOCX)

S2 Table Carbon dioxide-anesthesia under water oxygenated with ultrafine bubbles. * Carbon dioxide concentration judged by macroscopic observation when it was in the anesthetic condition that the condition of the fish was equivalent to for the second phase from the first phase of the depth of anesthesia in human general anesthesia.
(DOCX)

S3 Table Relationship with the bubble diameter and the rising rate of a bubble in water. Stokes' law is as follows: [v_s_ = D_p_^2^ (σ_p_−σ_f_) g] / 18η v_s_: Terminal velocity [cm/s], D_p_: Particle diameter [cm], σ_p_: Particulate density [g/cm^3^], σ_f_: Density of the fluid [g/cm^3^], g: Acceleration of gravity [cm/s^2^], η: Coefficient of viscosity of the fluid [g/(cm·s)].
(DOCX)

S4 Table Relationship with the bubble diameter and the pressure in the bubble. Young-Laplace equation is as follows: ΔP = 4σ/d ΔP: Degree of the upward pressure [atm], σ: Surface tension [mN/m], d: Diameter of bubble [mm].
(DOCX)

## References

[1] Gomes LC, Araujo-Lima CARM, Chippari-Gomes AR, Roubach R. Transportation of juvenile tambaqui (*Colossoma macropomum*) in a closed system. Braz J Biol. 2006;66(2A): 493–502. 16862304 10.1590/s1519-69842006000300015

[2] Collymore C, Tolwani A, Lieggi C, Rasmussen S. Efficacy and safety of 5 anesthetics in adult zebrafish (*Danio rerio*). J Am Assoc Lab Anim Sci. 2014;53(2):198–203. 24602548 PMC3966278

[3] Chen K, Wang CQ, Fan YQ, Xie YS, Yin ZF, Xu ZJ, et al The evaluation of rapid cooling as an anesthetic method for the zebrafish. Zebrafish. 2014;11(1): 71–75. 10.1089/zeb.2012.0858 24093489

[4] Blessing JJ, Marshall JC, Balcombe SR. Humane killing of fishes for scientific research: a comparison of two methods. J Fish Biol. 2010;76(10): 2571–2577. 10.1111/j.1095-8649.2010.02633.x 20557609

[5] Mitsuda H, Ueno S, Mizuno H, Ueda T, Fujikawa H, Nohara T, et al Levels of CO2 in arterial blood of carp under carbon dioxide anesthesia. J Nutr Sci Vitaminol (Tokyo). 1982;28(1): 35–39. 6808097 10.3177/jnsv.28.35

[6] Chalon J, Martin P, Roberts C, Ramanathan S, Katz R, Turndorf H. Anaesthetic uptake by the goldfish: effect of respiratory rate. Acta Anaesthesiol Scand. 1983;27(5): 361–365. 6416014 10.1111/j.1399-6576.1983.tb01968.x

[7] Grottum JA, Sigholt T. Acute toxicity of carbon dioxide on European seabass (*Dicentrarchus labrax*): Mortality and effects on plasma ions. Comp Biochem Physiol. 1996;115(4): 323–327.

[8] Mi H, Qian C, Mao L. Quality and biochemical properties of artificially hibernated crucian carp for waterless preservation. Fish Physiol Biochem. 2012;38(6): 1721–1728. 10.1007/s10695-012-9669-2 22688451

[9] Mitsuda H, Yoshizawa H. Application of Cold-CO_2_-anesthesia for the Transportation of Live Fish. Jpn J Freezing and Drying. 1991;37: 54–60.

[10] Kugino K. Anesthesia methods of fish and shellfish and apparatus. International Applications under the Patent Cooperation Treaty Patent. 2014;PCT/JP2014/053673.

[11] Watanabe K, Takahashi M, Nakagawa M, Ohta K, Satoh J, Hotta T. Effectiveness of 2-phenoxyethanol anesthesia of fish cultured in Japan Aquaculture Sci. 2006;54(3): 255–63.

[12] Takeda T, Itazawa Y. Possibility of Applying Anesthesia by Carbon Dioxide in the Transportation of Live Fish. Bulletin of the Japanese Society of Scientific Fisheries. 1983;49(5): 725–731.

[13] Watanabe K. Anesthesia of Japanese flounder Paralichthys olivaceus juveniles by effervescent CO2-evolving tablet. Nippon Suisan Gakkaishi 2007;73(2): 287–289.

[14] Talley BD, Min DH, Lobanov VB, Luchin VA, Ponomarev VI, Salyuk An, et al Japan/east sea water masses and their relation to the sea’s circulation. Oceanography. 2006;19(3): 32–49.

[15] Stokes GG. On the theories of the internal friction of fluids in motion, and of the equilibrium and motion of elastic solids. Trans Camb Phil Soc, vol. 8 1845 p. 287–319.

[16] Young T. An essay on the cohesion of fluids. Philos Trans R Soc London. 1805;95: 65–87.

[17] Laplace PS. Traite de Mechanique Celeste: Supplements au Livre X. Gauthier-Villars, Paris: 1805. p. 349–498.

