# Supplementary material for: Long-Duration Carbon Dioxide Anesthesia of Fish Using Ultra Fine (Nano-Scale) Bubbles
Source: PLoS One. 2016 Apr 21;11(4):e0153542. doi: 10.1371/journal.pone.0153542 (PMC4839645; doi:10.1371/journal.pone.0153542)
Supplement: S1 Table — * Carbon dioxide concentration judged by macroscopic observation when it was in the anesthetic condition that the condition of the fish was equivalent to for the second phase from the first phase of the depth of anesthesia in human general anesthesia. (DOCX) [file pone.0153542.s001.docx]

**S1 Table. The time limits for conventional carbon dioxide-anesthesia for various aquatic species.** This is the Table 2 legend.

* Carbon dioxide concentration judged by macroscopic observation when it was in the

anesthetic condition that the condition of the fish was equivalent to for the second phase from the first phase of the depth of anesthesia in human general anesthesia.

**Table 2. The time limits for conventional carbon dioxide-anesthesia for various aquatic species**

| Fish species | Individual number | CO_2_ level for anesthesia initiation* (%) | CO_2_ level for maintenance of anesthesia (%) | Lethal time　(min.) |
| --- | --- | --- | --- | --- |
| Bigfin reef squid | No.1 | 5.3 | 7.0 | 10 |
|  | No.2 | 4.2 | 7.0 | 15 |
| White-lined rockcod | No.3 | 6.8 | 8.0 | 10 |
|  | No.4 | 5.8 | 8.0 | 20 |
| Chicken grunt | No.5 | 4.0 | 5.0 | 10 |
|  | No.6 | 2.9 | 5.0 | 10 |
| Japanese scad | No.7 | 4.3 | 7.0 | 10 |
|  | No.8 | 6.0 | 7.0 | 15 |
| Red seabream | No.9 | 8.9 | 10.0 | 25 |
|  | No.10 | 7.3 | 10.0 | 15 |
