# Supplementary material for: Long-Duration Carbon Dioxide Anesthesia of Fish Using Ultra Fine (Nano-Scale) Bubbles
Source: PLoS One. 2016 Apr 21;11(4):e0153542. doi: 10.1371/journal.pone.0153542 (PMC4839645; doi:10.1371/journal.pone.0153542)
Supplement: S2 Table — * Carbon dioxide concentration judged by macroscopic observation when it was in the anesthetic condition that the condition of the fish was equivalent to for the second phase from the first phase of the depth of anesthesia in human general anesthesia. (DOCX) [file pone.0153542.s002.docx]

**S2 Table. Carbon dioxide-anesthesia under water oxygenated with ultrafine bubbles.** This is the Table 3 legend.

* Carbon dioxide concentration judged by macroscopic observation when it was in the

anesthetic condition that the condition of the fish was equivalent to for the second phase from the first phase of the depth of anesthesia in human general anesthesia.

**Table 3. Carbon dioxide-anesthesia under water oxygenated with ultrafine bubbles**

| Fish species | Individual number | CO_2_ level at anesthesia initiation* (%) | CO_2_ level at the maintenance of anesthesia (%) | CO_2_ level at anesthesia awakening (%) |
| --- | --- | --- | --- | --- |
| Chicken grunts | No.1 | 2.8 | 5.0 | 1.2 |
|  | No.2 | 3.8 | 5.0 | 1.8 |
|  | No.3 | 3.0 | 5.0 | 1.2 |
|  | No.4 | 3.4 | 5.0 | 1.2 |
|  | No.5 | 4.2 | 5.0 | 2.0 |
