# Supplementary material for: Long-Duration Carbon Dioxide Anesthesia of Fish Using Ultra Fine (Nano-Scale) Bubbles
Source: PLoS One. 2016 Apr 21;11(4):e0153542. doi: 10.1371/journal.pone.0153542 (PMC4839645; doi:10.1371/journal.pone.0153542)
Supplement: S3 Table — Stokes' law is as follows: [vs = Dp2 (σp−σf) g] / 18η vs: Terminal velocity [cm/s], Dp: Particle diameter [cm], σp: Particulate density [g/cm3], σf: Density of the fluid [g/cm3], g: Acceleration of gravity [cm/s2], η: Coefficient of viscosity of the fluid [g/(cm·s)]. (DOCX) [file pone.0153542.s003.docx]

**S3 Table. Relationship with the bubble diameter and the rising rate of a bubble in water.** This is the Table 4 legend.

Stokes' law is as follows.

[v_s_ = D_p_^2^ ( σ_p_ – σ_f_ )g ] / 18η

v_s_ : Terminal velocity [cm/s], D_p_ : Particle diameter [cm], σ_p_ : Particulate density [g/cm^3^], σ_f_ : Density of the fluid [g/cm^3^], g : Acceleration of gravity [cm/s^2^], η : Coefficient of viscosity of the fluid [g/(cm･s)].

**Table 4. Relationship with the bubble diameter and the rising rate of a bubble in water**

| Diameter of bubble | Rising rate of bubble in water (v_s_) |
| --- | --- |
| 100 μm | 5440 μm/s |
| 10 μm | 54.4 μm/s ≒ 19.6 cm/h |
| 1 μm | 0.544 μm/s ≒ 2.0 mm/h |
