# Supplementary material for: Long-Duration Carbon Dioxide Anesthesia of Fish Using Ultra Fine (Nano-Scale) Bubbles
Source: PLoS One. 2016 Apr 21;11(4):e0153542. doi: 10.1371/journal.pone.0153542 (PMC4839645; doi:10.1371/journal.pone.0153542)
Supplement: S4 Table — Young-Laplace equation is as follows: ΔP = 4σ/d ΔP: Degree of the upward pressure [atm], σ: Surface tension [mN/m], d: Diameter of bubble [mm]. (DOCX) [file pone.0153542.s004.docx]

**S4 Table. Relationship with the bubble diameter and the pressure in the bubble.** This is the Table 5 legend.

Young-Laplace equation is as follows.

∆P = 4σ/d

∆P : Degree of the upward pressure [atm], σ : Surface tension [mN/m], d : Diameter of bubble [mm].

**Table 5. Relationship with the bubble diameter and the pressure in the bubble**

| Diameter of bubble | Pressure in the bubble in water |
| --- | --- |
| 1 mm | 1.003 atm |
| 100 μm | 1.03 atm |
| 10 μm | 1.29 atm |
| 1 μm | 3.9 atm |
| 500 nm | 5.8 atm |
| 300 nm | 9.7 atm |
| 200 nm | 14.6 atm |
| 100 nm | 29.7 atm |
